# Laboratory capacity assessments in 25 African countries at high risk of yellow fever, August-December 2018

**DOI:** 10.11604/pamj.2021.38.402.28886

**Published:** 2021-04-27

**Authors:** Barbara Wilmot Johnson, Maurice Demanou, Gamou Fall, Jean-Luc Betoulle, Celestina Obiekea, Alison Jane Basile, Cristina Domingo, Christin Goodman, Eric Mossel, Chantal Reusken, Erin Staples, Joana Filipa Machado de Morais, Zoraima Neto, Paula Paixão, Yves Eric Denon, Mariette Glitho, José Mahinou, Therese Kagone, Emmanuel Nakoune, Kadidja Gamougam, Elisabeth Pukuta Simbu, Steve Ahuka, Jean-Vivien Mombouli, Cynthia Goma-Nkoua, Edgard Valery Adjogoua, Adamu Tayachew, Berhane Beyene, Bakary Sanneh, Modou Lamin Jarju, Alphonse Mendy, Dodzi Kofi Amelor, Lawrence Ofosu-Appiah, David Opare, Lorreta Antwi, Rexford Adade, N'Faly Magassouba, Sabado Fernandes Gomes, Samson Limbaso, Joel Lutomiah, Burgess Gbelee, John Dogba, Issa Cisse, Zakou Idde, Chikwe Ihekweazu, Nwando Mba, Ousmane Faye, Oumar Faye, Amadou Alpha Sall, Zikan Koroma, Manuela Alphonse Juma, James Ayei Maror, Mawahib Eldigail, Adel Hussein Elduma, Rehab Elageb, Kossi Badziklou, Koba Adjaho Komla, John Kayiwa, Julius Julian Lutwama, Lee Hampton, Mick Norman Mulders

**Affiliations:** 1 Scientific Laboratory Consulting, Laporte, Colorado, United States of America,; 2 World Health Organization African Region Yellow Fever Laboratory, Ouagadougou, Burkina Faso,; 3 Centre Pasteur Cameroon, Yaoundé, Cameroon,; 4 Institut Pasteur Dakar, Dakar, Senegal,; 5 Scientific Laboratory, Atlanta, Georgia, United States of America,; 6 National Reference Laboratory, Nigeria Centre for Disease Control, Abuja, Nigeria,; 7 Centers for Disease Control and Prevention, Division of Vector-borne Diseases, Fort Collins, Colorado, United States of America,; 8 Robert Koch Institute, Berlin, Germany,; 9 Centre for Infectious Disease Control, National Institute for Public Health and the Environment, Bilthoven, The Netherlands,; 10 Instituto Nacional de Investigacao em Saude, Luanda, Angola,; 11 National Public Health Laboratory, Cotonou, Benin,; 12 Centre Muraz, Bobo Dioulasso, Burkina Faso,; 13 Institute Pasteur, Bangui, Central African Republic,; 14 Hôpital General De Reference Nationale, N'Djamena, Chad,; 15 National Institute for Biomedical Research, Kinshasha, Democratic Republic of Congo,; 16 Institut National de Santé Publique, Brazzaville, Republic of Congo,; 17 Institut Pasteur de Côte d'Ivoire, Abidjan, Côte d'Ivoire,; 18 Ethiopian Public Health Institute, Virology and Rickettsiology Unit, Addis Ababa, Ethiopia,; 19 World Health Organization Laboratory, Ethiopia,; 20 National Health Laboratory Services, Royal Victoria Teaching Hospital, Banjul, The Gambia,; 21 National Public Health and Reference Laboratory, Public Health Division, Ghana Health Service, Korle-Bu, Accra, Ghana,; 22 Laboratoire de Reference des Fièvres Hémorragiques, Conakry, Guinée,; 23 Laboratoire National de Sante Publique, Bissau, Guinea-Bissau,; 24 Center for Virus Research, Kenya Medical Research Institute, Nairobi, Kenya,; 25 National Public Health Reference Laboratory, National Public Health Institute of Liberia, Charlesville, Liberia,; 26 Institut National de Santé Publique Laboratoire de Fièvre Jaune, Bamako, Mali,; 27 Niamey National Hospital, Niamey, Niger,; 28 National Public Health Laboratory, Lakka, Sierra Leone,; 29 National Public Health Laboratory, Juba, South Sudan,; 30 Public Health Laboratory, Khartoum, Sudan,; 31 Laboratoire de Sérologie, Institut National d'Hygiène, Lomé, Togo,; 32 Uganda Virus Research Institute, Entebbe, Uganda,; 33 Gavi Secretariat, Geneva, Switzerland,; 34 Department of Immunizations, Vaccines and Biologicals, World Health Organization, Geneva, Switzerland

**Keywords:** Yellow fever, laboratory, diagnostics, testing

## Abstract

**Introduction:**

accurate and timely laboratory diagnosis of yellow fever (YF) is critical to the Eliminate Yellow Fever Epidemics (EYE) strategy. Gavi, the Vaccine Alliance recognized the need to support and build capacity in the national and regional laboratories in the Global YF Laboratory Network (GYFLN) as part of this strategy.

**Methods:**

to better understand current capacity, gaps and needs of the GYFLN laboratories in Africa, assessments were carried out in national and regional reference laboratories in the 25 African countries at high risk for YF outbreaks that were eligible for new financial support from Gavi.

**Results:**

the assessments found that the GYFLN in Africa has high capacity but 21% of specimens were not tested due to lack of testing kits or reagents and approximately 50% of presumptive YF cases were not confirmed at the regional reference laboratory due to problems with shipping.

**Conclusion:**

the laboratory assessments helped to document the baseline capacities of these laboratories prior to Gavi funding to support strengthening YF laboratories.

## Introduction

Yellow fever (YF) virus is a mosquito-borne flavivirus that causes disease ranging from a mild fever to severe illness characterized by hemorrhagic fever, with a 30% - 60% fatality rate for the 15% who develop severe disease [1]. Yellow fever occurs in sub-Saharan Africa and tropical South America, where it is endemic and intermittently epidemic [2]. The strategies for YF prevention are personal protection against mosquito bites and community vector control, routine infant immunization for children aged 9 months or above, preventive and reactive mass vaccination campaigns aimed at wider age ranges and early outbreak detection and investigation [3].

Accurate, timely laboratory testing is critical for identifying YF cases and initiating the appropriate response for efficient outbreak control. Delays in YF laboratory and case confirmation in the 2015-2016 outbreak in Angola were shown to contribute to spread of YF into the Democratic Republic of Congo and exportation to China [4-7]. These YF outbreaks highlighted the need for better prevention, detection and response and led to the development and implementation of the global Eliminate Yellow Fever Epidemics (EYE) strategy, which emphasizes protecting populations at-risk for YF, preventing international spread of YF and containing outbreaks rapidly [8-10].

Laboratory confirmation of suspected YF cases is critical for effective surveillance and for guiding EYE efforts to prevent and control YF [11,12]. Clinical diagnosis of YF, particularly of an isolated case in an area of low incidence, is difficult because symptoms, characterized by acute onset of fever followed by jaundice and hemorrhagic fever in severe cases, may be similar to those of many other diseases, such as viral hepatitis, malaria, dengue, typhoid fever, leptospirosis and other viral hemorrhagic fevers (VHFs). In addition, a laboratory developed YF IgM antibody capture enzyme-linked immunosorbent assay (MAC-ELISA) with in-house produced reagents has been the primary test used and antigens in the assay can cross-react with antibodies to other flaviviruses co-circulating in the region and cause false-positive results [9,13,14]. Therefore, confirmatory differential diagnostic and/or molecular testing of specimens with YF-positive IgM results is an essential component of the YF testing algorithm [15].

The World Health Organization (WHO) Global YF Laboratory Network (GYFLN) was modelled after the Global Measles/Rubella (M/R) Laboratory Network (GMRLN), a tiered structure with the Global Specialized Laboratory (GSL) providing support to the Regional Reference Laboratories (RRLs), which in turn provide technical support, training and quality control (QC) and referral testing to the National Laboratories (NLs) [16]. Laboratories coordinate activities closely with national surveillance and Expanded Program for Immunization (EPI) programs. Additionally, the GYFLN RRLs must conduct confirmatory testing for specimens tested in NLs with YF IgM-positive or -equivocal (EQ) results and due to the lack of a validated commercial YF MAC-ELISA kit, produce and distribute reagents to NLs.

**Figure 1:**
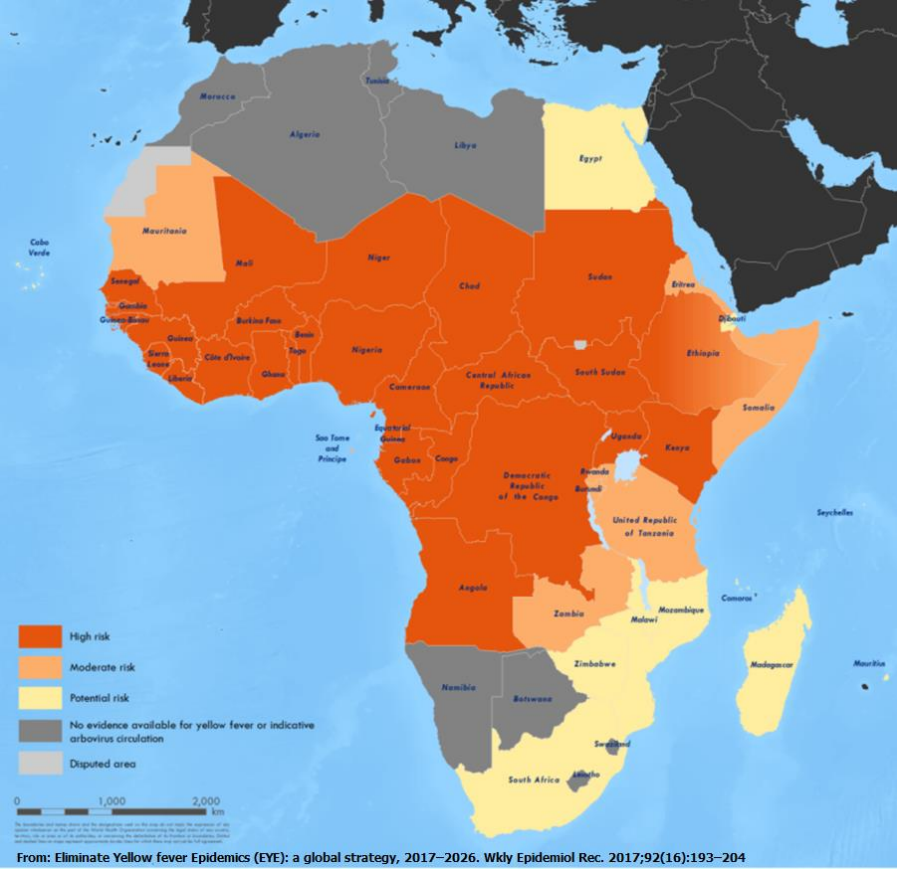
yellow fever risk classification in Africa by country in 2016; twenty-seven countries were identified as high risk for YF epidemics based on timing and intensity of YF virus circulation in the country, estimates of transmission potential, and assessment of urban outbreak risk; laboratory assessments were conducted in 25 YF NLs in high-risk countries eligible for Gavi support at the time of the assessments (excluded Equatorial Guinea and Gabon) and the RRL at IPD

Currently, much of Africa is considered at high risk for YF outbreaks and there is potential risk of YF transmission to other areas where the *Aedes* mosquito vectors occur [9]. Gavi, the Vaccine Alliance recognized the need to support and build capacity in the NLs and RRLs as part of the EYE strategy. Therefore, to better understand current capacities, gaps and needs of the GYFLN laboratories in Africa, assessments were carried out from August to December 2018, in 25 of the 27 African countries at high risk for YF outbreaks that were eligible for new financial support from Gavi (Figure 1) [17,18]. The assessments helped to document the baseline capacities of these laboratories prior to utilizing Gavi funding to support strengthening YF laboratories.

## Methods

On-site visits were conducted in 23 of the 25 laboratories assessed. Prior to the visits, information was collected through a questionnaire based on the YF performance indicators (PIs) in the WHO African region (AFR) laboratory accreditation form and checklist; key parameters used in the assessments are shown in Table 1. The term qualified was used in the assessment for NLs that conducted YF MAC-ELISA testing, whether or not they were accredited or proficient, as not all NLs had been reviewed [15]. National Laboratories without capacity included those without an adequate serological laboratory, without trained laboratory personnel, or non-proficient. Nucleic acid testing (NAT) capacity at NLs was also assessed to indicate the potential for implementing molecular testing into the testing algorithm in Africa [15].

**Table 1:** key laboratory capabilities assessed^a^

Assessment category	Performance measurement
Equipment/facility	Functioning equipment
	Routine preventive maintenance and/or calibration
	Routine certification of BSCs
	Laboratory clean and uncluttered with appropriate separation of activities
	Reliable source of electricity and/or backup generator
Laboratory personnel	Staff knowledgeable about GLP
	Well-trained and competent to conduct MAC-ELISA testing
	Trained in YF-specific laboratory techniques
Data management	Specimens received in laboratory with accompanying case investigation form
	Specimen number in laboratory logbook linked to case investigation number
	Laboratory testing and reporting completed within 7 days of specimen receipt
	Designated data manager responsible for entering results into Epi Info database
	Data routinely harmonized between agencies (MoH and WHO)
	Specimens stored appropriately with accurate inventory system
Procurement	Maintenance of accurate reagent inventory
	Efficient procurement system
YF testing	NL qualified^b^ to test specimens by YF MAC-ELISA
	All specimens tested within 7 days of receipt
	All specimens with YF IgM EQ results retested
	All specimens with YF IgM+ results sent to RRL for confirmatory testing
	All specimens with YF IgM EQ results after retesting sent to RRL for confirmatory testing
	Final confirmatory test results reported by RRL within 21 days of specimen receipt
	Results concordant with RRL results
Quality assurance	Written QA policy
	Designated laboratory QA manager
	SOPs documented and followed
	Document control system in place
	Monitoring and charting of temperature-sensitive equipment
	Use of internal control specimens
	External quality assurance programs (WHO GYFLN, SLIPTA, SLMTA, ISO)
Quality control	10% of specimens with YF IgM- results sent to RRL for QC retesting
	Results concordant with RRL results
Biosafety	Specimens processed in BSC
	Biohazard waste disposal system
Specimen transport	Specimens with YF IgM+/EQ results sent to RRL within 5 days after results available
	Specimens packed for shipping according to IATA standards
	Export/import permits and other documentation complete
	Package tracked during transport with notification of receipt by receiving laboratory
Molecular testing	Existing molecular laboratory in institute
	Appropriate separation of activities
	Laboratory staff trained in molecular testing procedures
	Existing DNA engines with open protocol programming

a Modified from the WHO African Region Yellow Fever National Laboratory Checklist for Annual WHO Accreditation; ^b^NL determined by WHO YF Laboratory coordinators to be qualified to conduct testing. Some of these NLs had not been reviewed for accreditation. Abbreviations: BSC: biosafety cabinet; DNA: deoxyribonucleic acid; EQ: equivocal; GLP: good laboratory practice; GYFLN: Global Yellow Fever Laboratory Network; IATA: International Air Transport Association; IgM: immunoglobulin M; ISO: International Organization for Standardization; MoH: ministry of health; QA: quality assurance; QC: quality control; RRL: Regional Reference Laboratory; SLIPTA: Stepwise Laboratory Improvement Process Towards Accreditation; SLMTA: Strengthening Laboratory Management Toward Accreditation; WHO: World Health Organization; YF: yellow fever; YF MAC-ELISA: YF IgM antibody capture enzyme-linked immunoassay

The questionnaire and all correspondence with the NLs and RRL were available in English, French and Portuguese. The visit consisted of an introduction of the assessment procedure in a briefing to laboratory staff, the relevant Ministry of Health (MoH) and WHO country representatives followed by a laboratory review, completion of the questionnaire with laboratory staff, interviews of laboratory staff and an exit briefing. A summary report for each country was reviewed by these stakeholders with corrections made as needed prior to submission of the final report to Gavi and WHO. An on-site assessment in Nigeria was not done in 2018 due to logistical challenges and security concerns for visiting the four NLs. Information on YF testing activities in Nigeria was obtained from the 2017 Nigeria Centre for Disease Control (NCDC) situation report and a report from a supervisory support visit to four Nigerian M/R and YF NLs by the NCDC laboratory coordinator from November 2018 to January 2019. The Uganda Virus Research Institute (UVRI) had been assessed in 2017 as part of a WHO accreditation review to recommend and designate UVRI as the second WHO African YF RRL; UVRI also provided updated information in the questionnaire.

A summary of YF testing activities from 2015-2017, collected from the individual questionnaires, is shown in Table 2. The 2017 data was chosen to detail YF testing activity because it was the most recent complete year for which data were available and most likely to accurately reflect current conditions, as the 2014-2016 Ebola outbreak had a significant effect on YF testing activities in 2015 and 2016 in many of these laboratories. The 2018 information was incomplete and therefore omitted. The Institut Pasteur de Dakar (IPD) was the sole YF RRL in AFR as well as the YF NL in Senegal. Test results from specimens submitted to IPD from the Senegal YF surveillance program were included with those of the other NLs. Test results of specimens sent from the NLs to the RRL at IPD for confirmatory and QC testing are shown separately in Table 2.

**Table 2:** YF testing activity 2015-2017 in 25 NLs and 1 RRL in Africa

	2015	2016	2017
**YF MAC-ELISA testing in NLs**			
# NLs that received specimens	19	21	23
Total # specimens received in NLs	7261	16,129	13,296
Total # specimens tested^a^ (% of received)	7023 (99)	15,646 (97)	10,456 (79)
**YF NAT in NLs**			
# NLs tested specimens by NAT (%)	4	7	7
# specimens tested by NAT (% #specimens received)	43 (0.6)	6740 (44)	2104 (16)
# NLs with NAT+ results	2	3	3
# specimens with NAT+ results (% of tested)	7 (16)	902 (13)	16 (0.8)
**RRL testing**			
Total specimens received from NLs	696	1271	745
# specimens received for QC testing	587	604	465
# specimens received for confirmatory testing	109	667	280
#specimens tested by NAT at RRL	78	689	242^b^
# specimens with NAT+ results at RRL (% of tested)	2 (3)	47 (7)	0 (0)

a Number of specimens tested includes those tested at qualified NLs and those received at NLs without testing capacity which were sent to the IPD NL or other qualified NL for primary testing; ^b^Number of specimens submitted for confirmatory testing with adequate volume for NAT. Abbreviations: EQ: equivocal; IgM: immunoglobulin M; MAC-ELISA: IgM antibody capture enzyme-linked immunosorbent assay; NL: national laboratory; NA: data not available; NAT: nucleic acid test; QC: quality control; RRL: regional reference laboratory; YF: yellow fever.

## Results

**Equipment and facility maintenance:** electricity outages occurred in 14 NLs and disrupted YF MAC-ELISA testing in nine of them (Table 3). Of the 14 NLs with backup electricity systems, eight had reliable backup generators; in the remaining six NLs, backup systems were unreliable (N=2), lacked fuel (N=1), did not turn on automatically (N=1), or had restricted access (N=2). Generators were the sole source of electricity in two NLs. Yellow fever MAC-ELISA testing was generally conducted in a shared serological laboratory space with equipment such as plate washers and readers used for both YF and M/R testing (Table 3). The serology laboratory space was described by the assessor or NL as inadequate in eight NLs, four of which conducted YF testing and four of which did not. NLs that routinely received many specimens per week set up schedules with designated days and times for YF testing, whereas those that received few YF specimens at a time stored them and tested them in batches, or when they received enough for one plate of specimens, but within the 7-day PI, if there were sufficient reagents. Four NLs did not have functioning plate washers and either washed the ELISA plates manually or used the washer in another laboratory. Three NLs did not have a functioning plate reader; two carried the plate to another laboratory room to complete the testing and one borrowed the equipment from another department. Three NLs reported that YF testing was interrupted due to ELISA equipment failure in 2017. In-country services providing equipment preventive maintenance and repair, biosafety cabinet (BSC) certification and pipette calibration were not available for most of the NLs; however, institution or government engineering departments did provide limited repair services (data not shown). Fifteen NLs had access to a BSC for processing specimens. The RRL had dedicated YF MAC-ELISA, plaque reduction neutralizing antibody test (PRNT), virus isolation and molecular laboratories, with functioning equipment and reliable electricity sources and backup generators.

**Table 3:** laboratory equipment and facilities in assessed NLs

	# laboratories		
Laboratory facility	Yes	No	Unknown
Electricity outages	14	10	1
Back-up generator	14	7	4
Testing interrupted due to electricity outage	9	15	1
Functioning autoclave or incinerator for biohazard waste disposal	17	7	1
**Serological laboratory**			
Laboratory space adequate	17	8	
Functioning ELISA plate washer	21	4	
Functioning ELISA plate reader	22	3	
ELISA testing interrupted due to equipment failure	3	22	
BSC in serology laboratory	15	10	
**Molecular laboratory**			
Molecular laboratory at institute	22	3	
Real-time DNA engine programmable for YF NAT	19	5^a^	1
YF molecular testing in NL	10	15	

a Three NLs had real-time DNA engines programed at the manufacturer; two NLs did not have instrumentation. Abbreviations: BSC: biosafety cabinet; NF: not functioning; ELISA: enzyme-linked immunosorbent assay; NAT: nucleic acid test; NL: national laboratory; YF: yellow fever.

**Laboratory personnel:** NL staff generally had been trained and received certifications at technical or academic institutes and was familiar with good laboratory practice (GLP). New staff was trained in the institute´s specific laboratory techniques by other members of the laboratory or through in-house or external training courses. Some NLs had strategies to cross-train technicians for all serologic and molecular techniques as preparation for outbreaks; other laboratories assigned specific testing responsibilities to individuals. In most NLs, technicians were responsible for both M/R and YF MAC-ELISA testing. According to the questionnaires, few NLs reported being understaffed and/or overburdened due to the high numbers of specimens received from surveillance programs and/or additional responsibilities for testing clinical or research project specimens. WHO AFR provided laboratory technical and material support and training workshops were often coordinated to include both M/R and YF, but there was comparatively limited training for YF-specific laboratory techniques. Five NLs reported that the WHO Laboratory Coordinators (LCs) and/or RRL staff provided YF training on-site; 4 NLs sent technical staff to the RRL for YF training; 13 NLs participated in at least one regional YF laboratory training workshop; and 7 NLs had no YF-specific laboratory training, including 2 NLs that did not conduct YF testing (data from questionnaire, not shown).

**Data management:** data management procedures were the same for M/R and YF surveillance. NLs had a designated data manager or trained laboratory technician who recorded results in their MoH YF surveillance database after review by the YF NL director. Confirmatory test results reporting was well established from the RRL to the respective WHO country and MoH offices and the information was entered into the Epi Info™ database, which is in the public domain and used by WHO, but two NLs did not have test result notifications from the RRL in their own records. Three NLs lacked coordination with the RRL. One country had no YF surveillance program and one NL lacked a YF specimen submission protocol (data not shown). Three NLs had an electronic specimen inventory system; two used Freezerworks™ and one used BioBank™ as part of its role as the Biological Resource Center within the West African Organization for Economic Cooperation and Development. NLs maintained inventories of archived specimens in a Microsoft Excel™ spreadsheet, although some inventory records lacked specimen locations. Two NLs did not have any specimen submission database or inventory system.

**Figure 2:**
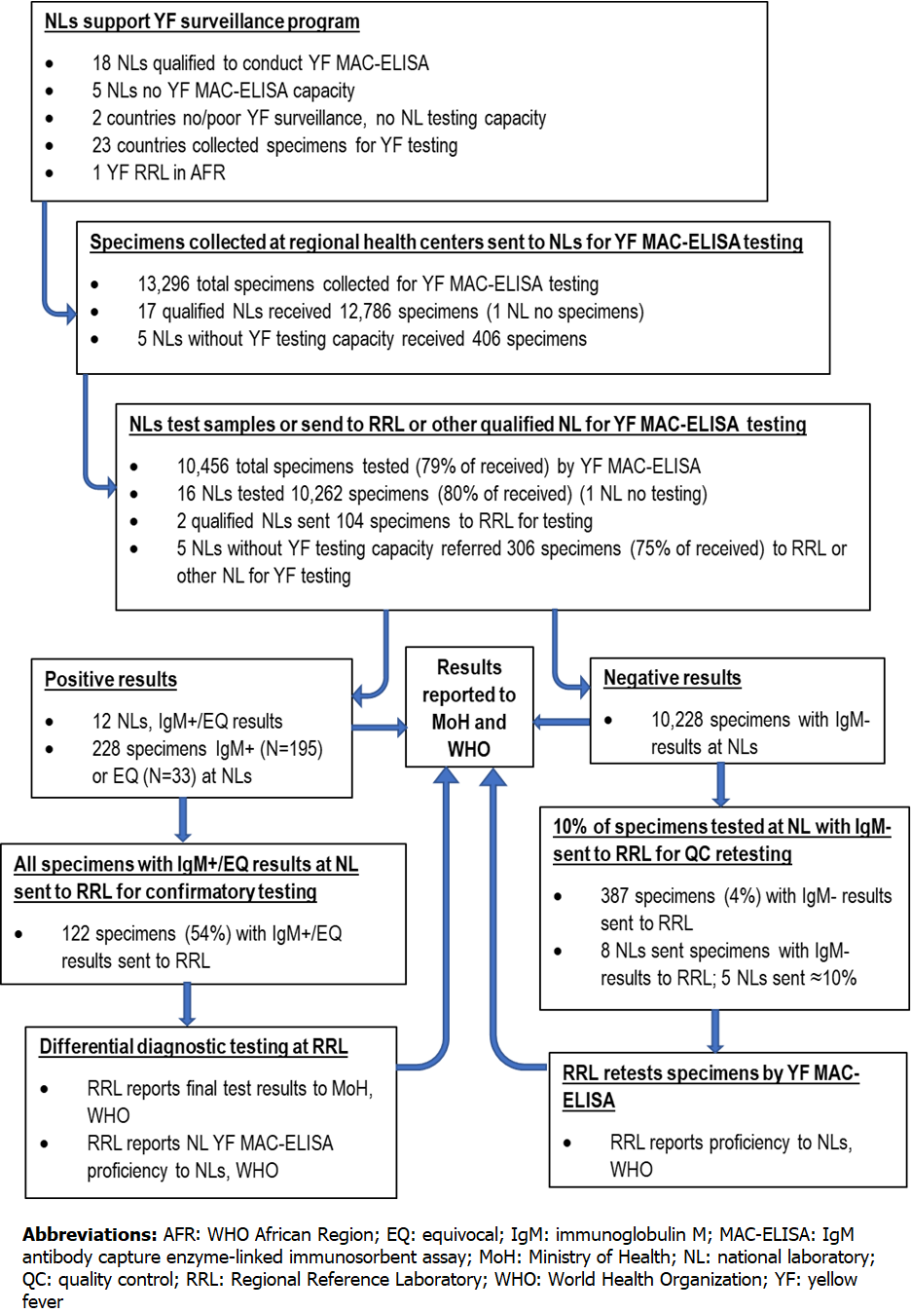
yellow fever serologic testing and referral algorithm in the WHO African Region with 2017 testing activities

**Procurement:** inventories of YF MAC-ELISA reagent and supplies were maintained in a Microsoft Excel spreadsheet or hard copy by a designated laboratory technician. A monthly inventory report was sent to the AFR LC in an email attachment. Requests for reagent resupply were also sent to the LC by email. Commercially available reagents and supplies were procured in bulk by WHO and fulfilled from WHO headquarters. Antigens, conjugate antibodies and positive control serum were produced by IPD or the US Centers for Disease Control and Prevention and distributed through WHO headquarters or by LCs. Because of shortages or stockouts of reagents or supplies, 12 of 17 NLs (71%) qualified to conduct YF MAC-ELISA testing lacked one or more critical reagents during part or all of 2017. Nine NLs could not test all specimens received, including one NL that could not test any received specimens due to lack of reagents throughout 2017. Two qualified NLs without sufficient reagents sent a portion of untested specimens to another NL or the RRL to test. Of the 2840 specimens not tested in 2017, 2740 (96%) were received in qualified NLs but not tested because the laboratory did not have the necessary reagents to run the YF MAC-ELISA (Figure 2). Reagents most reported out of stock were ELISA plates, antigens and substrate. Substitutions of substrate different from the SOPs were also reported.

**YF MAC-ELISA testing:** in 2017, 23 of the 25 NLs received a total of 13,296 specimens for YF testing in 2017, 10,456 (79%) of which were tested by YF MAC-ELISA (Table 2 and Figure 2). Eighteen NLs were considered qualified by the WHO LCs to test specimens (Figure 2). Of these, 17 qualified NLs received a combined total of 12,786 specimens for testing; one NL did not receive specimens for YF testing in 2017. Sixteen qualified NLs tested 10,262 specimens (78% of received) by YF MAC-ELISA. Nine qualified NLs could not test a total of 2740 specimens due to reagent stockouts, including one NL which did not test any of the 961 specimens it received. Two NLs sent 104 specimens to the RRL for primary testing. Five NLs without YF testing capacity collected a total of 406 specimens, 306 (75%) of which were referred to the RRL or other qualified NL for testing. One hundred specimens were not sent to another laboratory for YF testing due to shipping problems.

In 12 of the 15 NLs that tested specimens by YF MAC-ELISA, a total of 228 specimens had IgM-positive (N=195) or -EQ (N=33) results, 122 (54%) of which were sent to the RRL for confirmatory testing (Figure 2). Eight NLs reported that they sent all specimens with IgM-positive and -EQ results (N=112). One NL sent 10 of 20 specimens and two NLs did not send any specimens (N=87). Concordance of results between the NLs and RRL was 100% for 4 NLs, 57% for 1 NL and 30% for 1 NL according to the questionnaires; information on the final case classifications was not available. There was no correlation of results with the RRL for two NLs, one of which sent a single specimen with an IgM-EQ result and one of which sent two specimens. One NL did not have records of the test results from the RRL for the 8 specimens submitted. UVRI confirmed YF MAC-ELISA results itself by differential diagnostic MAC-ELISA testing, PRNT and NAT. The RRL reported that it received 280 specimens from NLs for confirmatory testing, all of which were tested unless they were received with insufficient volume or in poor condition, which included poor labeling, leaking or broken tubes, tubes with bacterial or fungal growth and contents not matching documentation. Of these, 240 had sufficient volume for NAT testing (Table 2).

**Quality assurance, quality control and biosafety:** WHO M/R and YF laboratory reviews were generally combined and used the same checklists and accreditation criteria. NLs were classified as accredited (N=8), partially accredited or pending (N=3), reviewed with the checklist (N=4), unsatisfactory (N=2), or had not been reviewed within the last 10 years (N=8) (Table 4). One accredited NL, at UVRI, also had been additionally evaluated for RRL designation in 2017. Eighteen NLs and the RRL were considered qualified to conduct YF MAC-ELISA testing, including three NLs without previous YF checklist reviews (Figure 2). Two of the five NLs without YF testing capacity had previous unsatisfactory ratings; they sent specimens to the NL in an adjoining country or the RRL for testing since 2012 and 2016, respectively.

**Table 4:** WHO YF laboratory accreditation and external QC/QA programs

WHO AFR	WHO YF checklist	WHO YF accreditation	Partially accredited or pending accreditation^a^	Unsatisfactory	None
	4	8	3	2	8^b^
**External QA**	**SLMTA**	**SLIPTA**		**ISO**	**None**
	1	10		3	15

a Partially, some deficiencies which if corrected would result in full accreditation; pending, site visit has been delayed or planned but not completed; ^b^Includes five countries not currently conducting YF testing. Abbreviations: AFR: African Region; SLMTA: Strengthening Laboratory Management Toward Accreditation; SLIPTA: Stepwise Laboratory Improvement Process Towards Accreditation; ISO: International Organization for Standardization; QA: quality assurance; WHO: World Health Organization; YF: yellow fever.

**Quality assurance (QA):** throughout the WHO laboratory networks, QA policy had been developed and QA managers, generally from the technical staff, had received training on procedures, but there was considerable variation, with some NLs having a formally designated, dedicated quality manager who drafted the QA policy to QA responsibilities added to the duties of laboratory technical staff, with no written policy. Information from the questionnaires was incomplete but it appeared that many of NLs still did not have written internal QA programs. QA weaknesses noted by assessors included use of standard operating procedures (SOPs) that had not undergone validation, nonadherence to SOPs, lack of use of in-house controls and monitoring and charting of assay controls, and gaps in daily monitoring of temperature-sensitive equipment. Ten NLs and the RRL participated in the external QA accreditation process Stepwise Laboratory Improvement Process Towards Accreditation (SLIPTA) (Table 4), with the RRL and two NLs additionally in progress towards International Organization for Standardization (ISO) accreditation. One NL was reviewed by the Strengthening Laboratory Management Towards Accreditation (SLMTA) program and working towards SLIPTA review. Fifteen NLs did not participate in any external QA program.

**Quality control:** unless the NL had been recently reviewed on-site, confirmatory retesting at the RRL of specimens tested in the NLs with IgM-positive results and 10% with IgM-negative results was the only QC PI to determine NL proficiency (Figure 2). Of the 10,228 specimens tested in the NLs with IgM-negative results, 387 specimens (4%) were sent to the RRL for QC retesting by 8 of the 16 NLs that conducted YF testing in 2017; 5 NLs sent at least the required 10%; 8 NLs did not send any specimens. The Senegal NL was co-located with the RRL at IPD and QC retesting of the 573 specimens with IgM-negative results was excluded from this analysis. Concordance between the RRL and NL results was as follows: 100% for 5 NLs (N=306), 94% for 1 NL (N=30) and 88% for 1 NL (N=51). Correlation between the RRL and these NLs was also high for specimens tested at the NLs with YF IgM-positive results.

**Biosafety:** staff generally was knowledgeable and had implemented basic safety and personal protective equipment rules in the laboratory. In 15 of 25 NLs (60%), specimens from YF suspected cases were processed in a BSC or glove box per WHO recommendations (Table 3). In two NLs where YF testing was part of VHF surveillance, the entire YF MAC-ELISA procedure was carried out in the BSC. One NL had a central reception department which heat-inactivated an aliquot of the specimen in a glove box prior to delivery to the laboratory. Seventeen of the 25 NLs had biohazard waste disposal procedures. Collection varied, from the laboratory personnel sealing the waste and delivering it to the autoclave/incinerator themselves to collection of biohazard waste in the laboratory by trained, dedicated staff (Table 3). Incineration was the primary method of biohazard waste disposal, with transport of waste to an off-site incinerator reported by one NL. There was limited availability and use of autoclaves due to inadequate and/or unreliable electricity or water. Nonfunctioning autoclaves were often not repaired or replaced due to lack of in-country preventive and repair services. Two of the seven NLs with no biohazard disposal equipment conducted YF MAC-ELISA testing.

**Figure 3:**
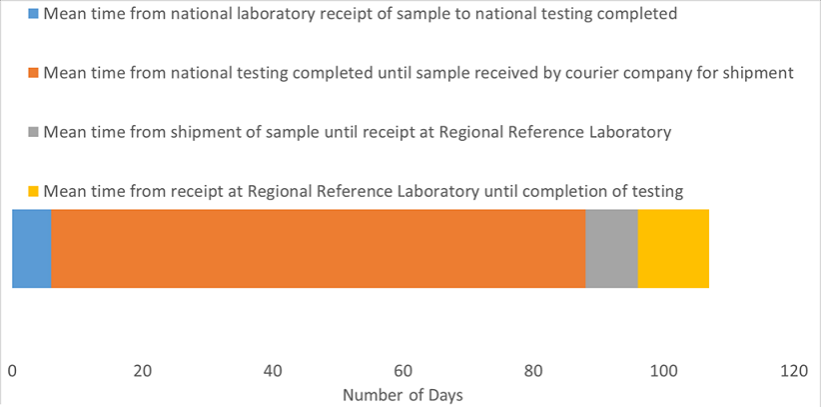
mean time from receipt of specimens in 14 NLs to confirmation at the RRL was 107 days during 2017; according to WHO performance indicators, specimens with YF IgM-positive or -EQ results were to be shipped to the RRL for confirmatory testing within 7 days of completion of testing; in 2017 the mean time from completion of testing at the NL to receipt of specimens by the courier was 82 days; note: Figure reflects data from Angola, Benin, Burkina Faso, Cameroon, Central African Republic, The Gambia, Ghana, Kenya, Mali, Niger, Nigeria, Sudan, and Togo

**Specimen transport:** specimens were transported from regional health centers to the WHO country office, MoH surveillance department, or directly to NLs through various mechanisms ranging from designated surveillance system vehicles, public buses, with shipments of specimens from other disease surveillance programs such as M/R and VHFs, or in batches when health center staff travelled to the city where the NL was located. Twelve NLs reported that they received specimens collected at regional health facilities within the 3-day PI (data not shown). Ten NLs reported delays in transport from regional health centers due to lack of funding for specimen transport, lack of reliable transport vehicles and long transport time from remote sites over long distances on roads in poor condition. Eight NLs reported receiving poor quality specimens due to hemolysis, poor specimen storage and leaking. Laboratory personnel noted disruption of the cold chain could have occurred during transport without their knowledge when specimens were not delivered directly to the laboratory. The mean time from receipt of a specimen at the NL to completion of testing was 6 days (PI =7 days; Figure 3).

There was at least one staff member with International Air Transport Association (IATA) dangerous goods training to pack specimens for international transport to the RRL in 20 of the 24 countries, either in the NL, MoH or other government department, or WHO country office. However, shipping specimens internationally to the RRL for confirmatory or QC testing was a continual problem, mainly due to lack of funding, but also because of inadequate documentation or couriers refusing to accept blood specimens, particularly after the Ebola outbreak. Shipments of specimens with YF IgM-positive or -EQ results that were shipped to the RRL in 2017 were delayed, primarily between the time from completion of testing at the NL to the courier receipt of the specimens (mean 82 days vs PI of 7 days), resulting in a mean total time of 107 days from when a specimen was received in the NL to confirmation at the RRL (Figure 3).

**Molecular testing capacity:** there were molecular testing facilities with functioning equipment and trained laboratory personnel in 22 NLs, primarily for polio, M/R, VHFs (including YF in some laboratories), influenza and human immunodeficiency virus (HIV) testing (Table 3). The ABI7500 thermocycler was the most common testing platform, listed by 14 NLs. Nine NLs reported that their equipment, materials and reagents, and instrument maintenance contracts had been donated for specific projects and in three NLs the thermocyclers were programmed for HIV testing only. Availability of RNA extraction and amplification kits was a limiting factor for YF molecular testing.

Ten NLs had incorporated molecular assays into their YF testing algorithm or conducted limited molecular testing for YF. Seven NLs tested a total of 2104 specimens (16% of received) by YF NAT in 2017, with 16 positive results among three NLs (Table 2). The RRL tested 242 specimens received from the NLs for confirmatory testing by NAT, with no positive results. Specimens tested by NAT were transported and processed with other serological specimens; there was no special handling to maintain the integrity of the viral ribonucleic acid (RNA). Criteria for YF NAT was a specimen collected <7 days post-onset of illness and sufficient volume remaining after serological testing [15]. There was no routine YF molecular testing QC program established in the NLs with the RRL.”. The protocols and primers/probe sequences used varied and included Domingo, unpublished in-house designed and commercial kits [19,20].

## Discussion

The GYFLN is modeled on the WHO global MRLN structure of tiered NLs and RRLs and strong coordination between the surveillance program and laboratory, well-established data management and results reporting systems which are integrated with other surveillance programs and a standardized system of specimen tracking [16]. Consequently, YF laboratory capacity was strongest in assessment categories for which protocols and performance measurements had been standardized for both the M/R and YF surveillance and laboratory programs, including specimen tracking systems, data management, results reporting, QA/QC programs and technical staff proficiency. Serology laboratory space, equipment and technical staff in NLs were shared between the M/R and YF laboratory activities and were generally adequate. Laboratory staff were trained in GLP and were technically skilled in YF MAC-ELISA, although there was limited YF-specific training, technical assistance, accreditation reviews, or proficiency testing programs and YF QC testing was limited to retesting specimens which had been tested in the NLs at the RRL.

The assessments illustrated two main barriers to timely and efficient laboratory diagnosis of YF that contributed to the lack of YF confirmed cases. First, because of frequent stockouts of one or more of the critical reagents needed to conduct the YF MAC-ELISA at the NLs, 21% of specimens collected from suspected YF cases were not tested in 2017. Second, because of problems with international shipping, only 54% of specimens with IgM-positive and -EQ results were sent to the RRL for confirmatory testing and those that were sent were significantly delayed while transport was being arranged. With a mean of 107 days from receipt at the NL to completion of confirmatory testing at the RRL, a YF outbreak could well have been underway before the first confirmed YF case was reported.

The findings from these laboratory assessments have guided efforts under the EYE strategy to address gaps and build YF laboratory capacity in Africa, with financial support from Gavi. Since the assessment, there have been two YF diagnostic laboratory workshops with participants from 33 high- and medium-risk African countries. Post-training serologic and molecular proficiency panels were distributed to participants to test in their own laboratories. Although promising, the results showed there is much room for improvement. Future workshops are anticipated to “train the trainers” at the regional level, with a biannual proficiency testing program to continually assess NL testing performance. Although there is presently no YF MAC-ELISA in kit format, numerous kit-based assays are in development and commercial companies and nonprofit organizations have recently shown interest in manufacturing YF kits. Guidelines for external validation of molecular and serologic kits are being finalized by the WHO Product Review Panel [21]. Until validated kits are available for distribution, Gavi is funding a reagent/component procurement and supply channel via United Nations Children's Fund (UNICEF) where eligible countries will be able to place orders through a centralized system for bundled components of the YF MAC-ELISA. This will increase YF testing availability and eliminate disruption of testing due to the lack of critical reagents. Bulk procurement will prevent the substitution of unvalidated reagents and significantly improve the quality of, and confidence in, the results.

There are now two additional RRLs in the region that can perform confirmatory testing, UVRI in Entebbe and the Centre Pasteur du Cameroon in Yaoundé. WHO has set up a contract with an international shipping company that arranges shipment of specimens from NLs to the RRLs. This should increase the proportion of specimens undergoing confirmatory testing and reduce the lag time for specimen transport, resulting in more complete and timely reporting of YF cases. Submission of specimens to the RRL for retesting is insufficient to thoroughly assess proficiency; therefore, a GYFLN-wide serology external quality assurance program is being organized by the EYE partners. Staffing for additional YF laboratory support and accreditation is being provided by the WHO. In addition, to ensure that equipment is functioning and equipment maintenance is ongoing, UNICEF will purchase extended warranties for new equipment and provide support for training of NL staff on equipment maintenance.

The EYE Laboratory Technical Working Group has developed a standardized, global YF testing algorithm with molecular testing added to the recommended AFR YF testing algorithm. When fully implemented, molecular testing of appropriately timed high-quality specimens should increase the number of YF cases confirmed in the NLs, thus reducing the time for reporting confirmed cases and the response time. Nigeria has already triggered multiple YF outbreak responses based on NAT results prior to confirmation at the RRL, during the critical early stages of the outbreaks [22]. Uganda has decided to apply for Gavi support introducing yellow fever vaccine into routine introduction in large part due to molecular and serology testing results from UVRI [23]. However, positive NAT results of a single blood specimen will still need to be confirmed in a RRL before further responses are initiated, as false-positive results can occur due to low prevalence of the disease as well as imperfect laboratory testing. Specimens with negative NAT results will still need to be tested by YF MAC-ELISA and the need to confirm cases positive by serology will remain. Building the YF molecular testing capacity in the NLs will require considerable resources to fund training, proficiency testing programs, technical support, provision of testing kits and equipment and maintaining a reverse cold chain or other system to preserve the clinical specimens during transportation. Sustaining molecular testing capacity will require support and investment by country MoHs.

There were limitations to these assessments. This report is based on information and data supplied in the Gavi questionnaire and during interviews at the laboratories, not official WHO or MoH reports. Descriptions of laboratory activity, procedures, coordination with EPI and problems with transportation were anecdotal from the laboratory point of view. The discrepancy in sample numbers tested between the NLs and the RRL is illustrative in Table 2. The national EPI database accessed from the laboratory was the source of most of the testing activity information. With few exceptions, surveillance officers were not part of the assessment interviews. Assessing whether laboratory procedures were followed was determined through records and logbooks as well as observation of monitoring and signoff sheets. On occasion, the NLs reviewing the draft reports disagreed with the problems and gaps noted by assessors and were removed from the final report, per prior agreement. In addition, the data collection methods used between the on-site assessments and those of Nigeria and Uganda varied. Finally, some countries at high risk for YF were not included in the assessment because their per capita gross domestic product was above the threshold for Gavi support. However, these NLs continue to receive support from WHO with the provision of reagents/supplies/equipment from the stockpile in WHO headquarters of MAC-ELISA test kits and technical support and training.

Building capacity in the GYFLN is essential for rapid detection of YF cases and planning the appropriate response. YF-specific funding from Gavi will address the gaps and strengthen the WHO laboratory network structure on which the GYFLN is built. The long-term goal is to make support for YF testing capacity financially sustainable and eventually transition financial responsibility to national governments.

## Conclusion

These assessments helped to document current capacity for YF testing and identify gaps and needs in NLs in 25 African countries at high risk for YF outbreaks.

### What is known about this topic

- Much of Africa is considered at high risk for YF outbreaks;
- Accurate and timely laboratory diagnosis of laboratory confirmation of suspected YF cases is critical for effective surveillance and for guiding the EYE strategy to prevent and control YF;
- The GYFLN structure of tiered NLs and RRLs and strong coordination between the surveillance program and laboratory is best suited for controlling YF.

### What this study adds

- The GYFLN in Africa has high capacity but there are gaps and problems that need to be addressed;
- Serum collected from suspected YF cases was often not tested in NLs due to reagent shortages;
- Many presumptive YF infections were not confirmed in the RRL due to problems with shipping.
